# Self-Reported Sleepiness after 2, 4, and 7 Consecutive Night Shifts and Recovery Days in Danish Police Officers

**DOI:** 10.3390/ijerph191710527

**Published:** 2022-08-24

**Authors:** Marie Aarrebo Jensen, Helena Breth Nielsen, Mikael Sallinen, Jesper Kristiansen, Åse Marie Hansen, Anne Helene Garde

**Affiliations:** 1 The National Research Centre for the Working Environment, 2100 Copenhagen, Denmark; 2 Finnish Institute of Occupational Health, 00032 Helsinki, Finland; 3 Section of Social Medicine, Department of Public Health, University of Copenhagen, 1353 Copenhagen, Denmark

**Keywords:** shift work, sleep, working hours

## Abstract

Background: Night shift work often implies shorter sleep duration and this can lead to sleepiness, which has been associated with an increased risk of accidents and injuries. The aim is to study how the number of consecutive night shifts affects self-reported sleepiness. Participants and methods: The study was a quasi-experimental, within-subject crossover study with 73 police officers. Three work schedules of two, four, and seven consecutive night shifts followed by the same number of recovery days, i.e., days worked or days off, was performed by all participants. Sleepiness was self-reported using the Karolinska sleepiness scale (KSS) every fourth hour on the last night shift and the last recovery day in each sequence. Results: We observed differences in the level of sleepiness between recovery days and night shift days but no differences in the pattern of sleepiness levels on night shift days in the different work schedules. The highest levels of KSS were observed before bedtime (at 07:00 after a night shift and 23:00 on a recovery day). Conclusion: The number of consecutive night shifts did not affect the self-reported levels of self-reported sleepiness among Danish police officers.

## 1. Introduction

In Europe it is estimated that 5–20% of the working population are involved in night work [1]. Night shift workers report reduced sleep, premature awakenings, and not getting enough sleep. In general, studies suggest a reduction in sleep duration by approximately 3 h following night shifts [2]. This means that employees, while working on night shifts, sleep far less than the 7 h per 24 h that is the minimum amount recommended for healthy adults [3,4].

The shorter sleep reported by employees working night shifts can lead to sleepiness. This can in turn lead to reduced performance and a higher risk of injury [5]. For example, experimentally-induced sleep restriction has been found to result in impairments in many cognitive domains [6,7]. Thus, it is not surprising that night work is indeed associated with occupational accidents [8]. A higher risk of injury was observed after evening and night work in the past week compared with only day work in a large cohort study of hospital and administrative workers [9].

Sleepiness has been shown to increase when working night shifts, and the degree of this increase may depend on the night shift schedule. Results from both experimental and observational studies show sleepiness is most profound on the first night shift [10,11,12,13,14,15,16,17]. In addition, working irregular shifts poses a risk of increased sleepiness and sleep debt, indicating an elevated need for recovery [18], which, in turn, has been found to be associated with an increased risk for diseases and suboptimal health [19,20]. Moreover, shift systems with several consecutive night shifts have been found to be associated more strongly with sleepiness than shift systems with fewer consecutive night shifts [21]. An increased risk of injuries was found with an increasing number of consecutive night shifts in a meta-analysis of eight studies and was highest on the fourth consecutive night shift [22]. A recent position paper recommended a maximum of three consecutive night shifts to reduce the risk of injuries [23].

Recovery from shift work is important in maintaining employee health and well-being [24,25], and sleep quality has been shown to be affected for up to two weeks after extended night work periods [26].

We hypothesize that self-reported sleepiness increases with the number of consecutive night shifts because of the build-up of sleep loss over each consecutive night shift day. Therefore, the objective of the present paper is to investigate self-reported sleepiness after 2, 4, and 7 consecutive night shifts with a corresponding number of recovery days in a quasi-experimental, within-subject crossover study among Danish male police officers in a real-life setting. More specifically, we addressed the following research questions:

1. Does self-reported sleepiness increase with an increasing number of consecutive night shifts?
2. Does the number of consecutive night shifts increase the risk of excessive levels of sleepiness?

The research questions were investigated in the three different schedules where sleepiness was measured during the day with the last night shift in a row and during the last day in the sequence of recovery days.

## 2. Materials and Methods

The project “In the Middle of the Night” supplied data for this paper. The project compared the effect of two, four, and seven consecutive night shifts on a range of health-related outcomes [27,28,29]. The present paper focuses on effects of sleepiness. The study was approved by The National Committee on Health Research Ethics in Denmark (protocol number H-4-2012-155).

### 2.1. Design

The study design was as a quasi-experimental, within-subject crossover study. The participants were exposed to three different work schedules: 2 night shifts followed by 2 recovery days (2+2), 4 night shifts followed by 4 recovery days (4+4), and 7 night shifts followed by 7 recovery days (7+7). Recovery days were defined as day shifts (31%) or days off (69%). Approximately 90% of participants had a day off on the first recovery day after a series of night shifts and 87% of recovery days in the 2+2 were days off, whereas 65% and 66% of recovery days in the 4+4 and 7+7 schedules, respectively, were days off.

Day shifts were shifts starting after 06:00 and ending before 21:00, typically from 07:00 to 15:00 (we allowed day shifts to end as late as 18:00), and night shifts were defined as at least 3 h between 23:00 and 05:00 and typically from 23:00 to 07:00 (we allowed the start of the shift to vary between 22:00 and 00:00). Seven days before starting a work schedule, the participants were not allowed to work night shifts. The police officers in this study were all experienced shift workers. The Danish police force do not have regular schedules, so number of consecutive night shifts vary, although they did not typically work seven consecutive shifts as part of their normal schedule. However, the police force and the police union agreed for participants in the study to have up to seven consecutive nights to help study the effects of night shifts. Even if is it not used commonly in the Danish Police force, it is a popular way of organizing shift work in other populations in Denmark.

The data were collected in April–June 2013 and September–November 2013. The three work schedules lasted 26 days in total. Each participant had longer than three months to complete the three work schedules, and each individual only participated in either the spring or the fall. Daylight saving time began 27 March and ended 27 October 2013, however none of the participants had data collection on these two dates.

The order of the three studied work schedules was neither fixed, nor random, but occurred as part of the normal scheduling to fit into the staffing needs of the police force. The police force was, however, instructed to plan the work schedules so they occurred in different orders if possible, which resulted in 28%, 27%, and 45% starting with 2+2, 4+4, or 7+7, respectively. The different work schedules occurred with minimum one week and maximum six weeks apart.

### 2.2. Recruitment Procedure and Study Population

Participation was approved by the police labour union and five police districts at Zeeland, Denmark, volunteered to participate. Inclusion criteria were non-smoking male police officers with night shifts as a part of their regular schedule. Recruitment was initiated with information meetings for the leaders, the people responsible for personnel-on-duty planning, and employee representatives. All potential participants in all districts received an email invitation to participate. In total, 121 police officers showed interest in participating in the study. Of these, 73 police officers completed at least one of the work schedules in this study.

### 2.3. Questionnaire

Information about night work experience, physical activity, self-rated overall health, general job satisfaction, and diurnal type was obtained via a background questionnaire. Diurnal type was assessed with a single question from the morningness–eveningness questionnaire (MEQ):

One hears about “morning” and “evening” types of people. Which one of these types do you consider yourself to be? (Response categories were: Definitely a “morning” type, Rather more a “morning” than an “evening” type, Rather more an “evening” than a “morning” type, Definitely an “evening” type).

### 2.4. Sleepiness Logs

The participants filled in a logbook every 4th hour when awake on the last night shift and the last recovery day in each work sequence. The participants were instructed to score sleepiness at fixed times: 07:00, 11:00, 15:00, 19:00, 23:00, and 03:00 if they were awake. They scored sleepiness using the Karolinska sleepiness scale (KSS) (1 = extremely alert, 2 = very alert, 3 = alert, 4 = rather alert, 5 = neither alert nor sleepy, 6 = some signs of sleepiness, 7 = sleepy, but no effort to keep awake, 8 = sleepy, some effort to keep awake, 9 = very sleepy, great effort to keep awake, fighting sleep. KSS is a 9-point Likert-type scale often used in scientific studies where people are asked to self-report how alert they feel right now. KSS scores of 7–9 correspond to rapidly increasing levels of sleepiness. Research has linked KSS scores to performance and other measures of fatigue in the workplace [30]. A KSS rating of 7 (“sleepy but not fighting sleep”) has been shown to be accompanied by physiological signs of sleepiness, such as occasional slow eye movements and elevated alpha and theta activity in the EEG1 [31].

### 2.5. Sleep

Sleep was measured using a combination of sleep diaries and actigraphy. Sleep quality and timing (including naps) was recorded upon awakening on all days in the three work schedules. Actiwatches (ActiGraph wGT3X-BT from ActiGraph, Pensacola, FL, USA) were worn on the non-dominant wrist during all days. To score sleep we used a sampling rate of 30 Hz and 1 min epochs. Data were analysed with ActiGraph Sleep Analysis (ActiGraph, Pensacola, FL, USA). In and out of bed times were taken from the sleep diaries. Detailed information on sleep measurements has been published previously [27].

### 2.6. Statistics

The statistical software SAS 9.3 (SAS Institute, Cary, NC, USA) was used in all statistical analysis. Regression was performed with the PROC MIXED procedure. Categorical variables were work schedules in three levels (2+2; 4+4; 7+7) and type of day in two levels (night shift; recovery days). A random intercept was used for each individual with a variance component covariance structure and a repeated statement for the KSS values with an autoregressive covariance structure. The time of day was categorized into seven categories at 07:00, at 11:00, at 15:00, at 19:00, at 23:00, and at 03:00.

To test for an interaction between work schedule and type of day (night shifts or recovery days) to test if the effect on sleepiness of the work schedules differed between night shifts and recovery days, we included all six measurement days (model 1).

A stratified analysis with night shifts and recovery days was used to test the effect of the work schedule. An interaction term between work schedule and time of day was included to test for differences in the levels of sleepiness between the work schedules. The model was adjusted for age. In a sensitivity analysis, diurnal type was included in the model as a categorical variable.

The odds ratio of having KSS of ≥7 (dichotomized) was estimated by using a logistic regression model (PROC GENMOD). The generalized estimating equations (GEE) method was included to account for the within-person associations. KSS were grouped into two categories KSS ≤ 7 or KSS > 6 in the logistic regression model.

## 3. Results

Table 1 describes the participants. The mean age of the participants was 38 (range 25–62), and 22% had less than three years of night shift work experience, 38% had three to ten years of night work experience, and 40% had more than ten years of night work experience. The participants had excellent or very good self-rated health, and they were physically active in their free time. Table 2 shows the mean total sleep time for the participants during the three different work schedules. Participants had shorter sleep after night shifts compared with recovery days. Total sleep time on the last night shift was shorter than on the other night shifts in each work schedule.

**Figure 1:**
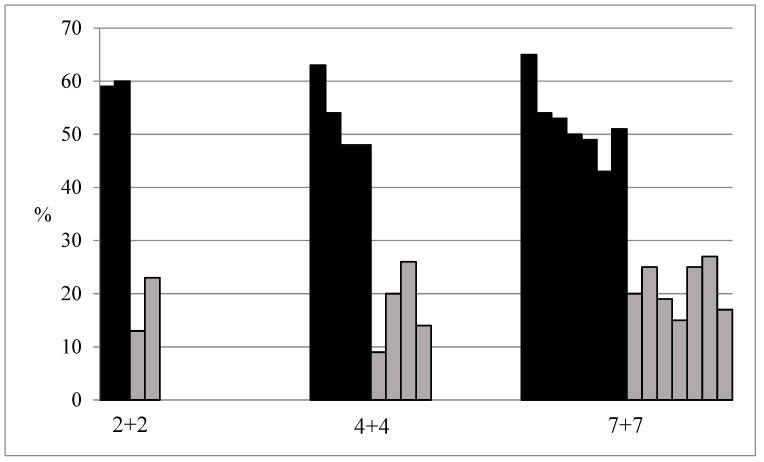
Distribution of napping in % of participants in the three different work schedules. Schedule 2+2 refers to 2 night shifts followed by 2 recovery days. Schedule 4+4 refers to 4 night shifts followed by 4 recovery days. Schedule 7+7 refers to 7 night shifts followed by 7 recovery days. Night shifts are shown in black and recovery days in grey.

Table 2 also shows the timing of average sleep onset during the three different work schedules and shows a relatively stable timing of sleep across the three different work schedules. The participants fell asleep around 08:00 on days with night shifts and around 23:45 on recovery days. The average time of awakening was 06:48 on recovery days with work and 07:25 on recovery days without work. The participants also used naps as part of their sleep behaviour. Most of the 73 participants had a nap during the three different work schedules, only one participant did not nap at all, and four participants solely napped on recovery days. Napping was more prevalent on days with night work (range: 43–65% of participants) compared to recovery days (range: 9–27% of participants). The number of naps per person on days with night work ranged from zero to three. The mean length of the first nap on the days with night work was 55.2 min (SD: 43.1 min). The mean length of second and third naps on days with night work was 53.2 (SD: 42.2 min). There seems to be a trend toward most participants taking a nap on the first night shift day in the 4+4 and 7+7 work schedule compared to the subsequent night shift days, see Figure 1.

However, this trend is not present in the 2+2 work schedule, where naps are approximately equally distributed on the two night shift days. Napping on restitution days seems to be more sporadically distributed with no particular tendency.

Table 3 shows the average KSS every fourth hour during the three different work schedules. The highest levels of KSS were observed at the end of the night shift and before bedtime on recovery days (at 07:00 after a night shift and 23:00 on a recovery day). The mean average (SD) KSS before bedtime ranged from 5.5 (2.6) to 6.0 (2.4) after night shifts and from 4.5 (2.4) to 5.1 (2.3) on recovery days. There were more KSS scores of 7 or higher on days with night shifts (22.8%) compared to recovery days (14.2%).

We found a significant interaction between the work schedules and type of day (night shifts or recovery days) for KSS (*p*-value for interaction ≤ 0.001). Therefore, stratified analysis with night shifts and recovery days was performed.

### 3.1. Recovery Days

**Figure 2:**
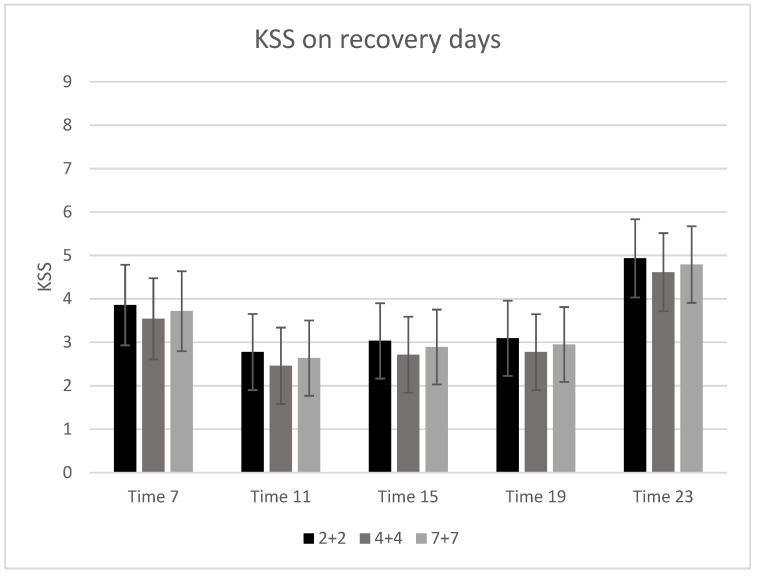

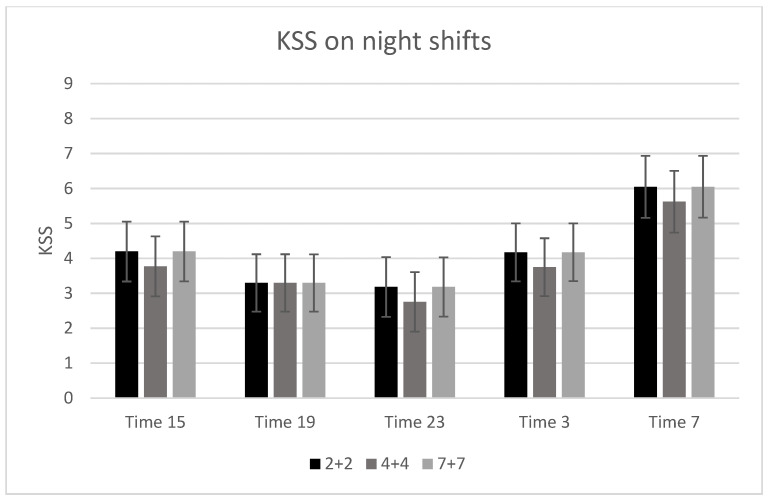
Estimates of KSS in the three different work schedules. KSS was measured every 4th hour at 07:00, 11:00, 15:00, 19:00, 23:00, and 03:00. Schedule 2+2 refers to 2 night shifts followed by 2 recovery days and is shown in black. Schedule 4+4 refers to 4 night shifts followed by 4 recovery days and is shown in dark grey. Schedule 7+7 refers to 7 night shifts followed by 7 recovery days and is shown in light grey. The error bars indicate 95% confidence interval.

The average values and the mean values of KSS at the five time points for the recovery days are presented in Figure 2. There was no significant difference between the work schedules in the levels of KSS (*p*-value for interaction = 0.333) on recovery days. Adjusting for diurnal type in the analysis did not affect the results (data not shown). There was no significant difference in the odds ratio of having a KSS of 7 or higher between the three different work schedules (*p*-value = 0.282).

### 3.2. Night Shifts Days

The average values and mean values of KSS for the night shift days can be seen in Figure 2. There was no significant difference between the work schedules in the levels of KSS (*p*-value for interaction = 0.298) on days with night shifts. Adjusting for diurnal type in the analysis did not affect the results (data not shown). There was no difference in the odds ratio of having a KSS of 7 or higher between the three different work schedules (*p*-value = 0.364).

## 4. Discussion

This study evaluated the sleepiness levels of 73 police officers working three different work schedules: “2 night shifts followed by 2 recovery days (2+2), 4 night shifts followed by 4 recovery days (4+4), and 7 night shifts followed by 7 recovery days (7+7)”. We found that participants did not report differences in sleepiness after two, four, and seven consecutive night shifts. We observed differences in the level of sleepiness between recovery days and night shift days. The highest levels of KSS were observed before bedtime (at 7:00 after a night shift and 23:00 on a recovery day). The mean (SD) KSS before bedtime ranged from 5.5 (2.6) to 6.0 (2.4) after night shifts and from 4.5 (2.4) to 5.1 (2.3) on recovery days. There were more KSS scores of 7 or higher after night shifts (22.8%) compared to recovery days (14.2%).

In a previously published study of these data, we found: “that sleep duration was reduced after night shift work and did not increase with more consecutive night shifts [27]. This showed that the cumulative sleep debt can be estimated as: (number of consecutive nights) × (difference between TST on night shift and recovery days) + (the difference between the last night shift in a series and other night shifts)”. The estimated cumulative sleep loss is 03:01, 05:09 and 08:21 h after two, four, and seven consecutive night shifts, respectively [27]. Here we show that despite the accumulated sleep loss, there was no increase in self-reported sleepiness with more consecutive night shifts. The differences found in accumulated sleep loss between the work schedules of the present study do not seem to be large enough to lead to differences in self-reported sleepiness in night working police officers. This is in line with a laboratory study showing that subjective sleepiness ratings showed an acute response to sleep restriction, but only small further increases on subsequent days in a study with 14 days of sleep restriction [32]. Sleep restriction in the study by van Dongen et al. resulted in cognitive performance deficits even though there were no changes in self-rated sleepiness. Lowden et al. also found no changes in KSS scores after four consecutive night shifts in a study of factory workers in the chemical industry [33].

In contrast to the lack of differences in self-reported sleepiness between the different work schedules in our study, van de Ven et al. found that ≥2 successive night shifts were related to more sleepiness than the first two night shifts in a study of shift workers in the entertainment industry [34]. Additionally, a Finnish study with questionnaire data linked to daily-based records of working hours found that short shift intervals and having >2 but not >4 consecutive night shifts were associated with increased odds of fatigue during work [18]. However, this was based on changes in work schedules over a longer period of time and may reflect more long-term or accumulated effects of having more consecutive night shifts compared to our study of acute effects.

Previous studies have consistently found the highest level of sleepiness on the first night shift [12,16], but since we were interested in consecutive night shifts, we did not measure sleepiness on the first night shift.

Almost all the participants in this study had naps during the three different work schedules. Napping has been found to sustain alertness and maintain performance [35,36], and the napping behaviour of the police officers could have reduced the level of sleepiness reported in this study.

Other studies among shift workers have also found excessive sleepiness during free days [37], suggesting that accumulated sleep deprivation during the work period may result in sleepiness on free days. We do not have a baseline measurement of sleepiness in this study as the aim was to compare the different work schedules. However, sleepiness during the recovery days did not differ, indicating the same level of recovery after night shift work even after seven consecutive work days. In this study, the ratio between the night shift days and the recovery days was constant. In addition, we only had measurements on the last day in each work schedule, and this may have contributed to the lack of differences between the recovery days.

The KSS is sensitive to fluctuations since it is a measure of situational sleepiness. Scores on the KSS strongly correlate with the time of the day and increase with increased periods of wakefulness. KSS measures acute sleepiness, not directly sleepiness accumulated over time. A police officer’s job may be quite stimulating and cause a high level of arousal. Arousal is known to influence self-reported sleepiness, and a high level of arousal masks the effects of sleep loss [38,39]. This phenomenon may partly explain the non-significant results. This means that one needs to be careful when generalizing the results to other types of occupations, especially with jobs that involve less arousal.

### Strengths and Limitations

The results of this study should be considered in the light of this study’s strengths and limitations. One possible limitation is that participants may be less likely to fill in the logbook if they are experiencing excessive sleepiness, resulting in a bias toward fewer indications of excessive sleepiness.

The participants in this study were all men. We believe that the underlying physiological mechanisms are similar in women, but men and women rate their sleepiness differently, with higher levels for women [40]. Accordingly, care should be taken when extending the results to groups of women.

The focus of this study was on the acute effects and not the long-term effects of shift work scheduling. Accordingly, each work schedule was only performed once. Since the effects of night shift work on sleepiness are considered both acute and reversible, we argue that the main potential differences between the three different work schedules in relation to sleepiness are best studied with the current design.

Compared with previous field studies, this study has a relatively large number of participants. The combination of the large number of participants and the quasi-experimental (rather than observational) crossover design, taking both within and between-subject variation into account in the statistical analyses, is a major strength. Internal validity is enhanced by circumventing potential confounding from, e.g., lifestyle factors. Crossover designs have some general limitations such as possible order effects and carry-over between the different work schedules. This was handled by mixing the work schedules so they occurred in different orders and using a 7-day period without night shifts before each work schedule.

The study used KSS, which is a validated and extensively used method of measuring sleepiness. KSS has been found to be closely related to electroencephalographic (EEG) activity and behavioral variables, indicating a high validity in measuring sleepiness [41].

The diurnal pattern of sleepiness is U-shaped, with high KSS scores in the morning and late evening [30]. We find this U-shape on recovery days on all three different work schedules. In addition, we find a similar U-shape on days with night shifts with high KSS scores after awakening and before bedtime. This result supports the face validity of the measurements carried out in the everyday life of police officers.

## 5. Conclusions

The self-reported sleepiness differed between days with night shift work and recovery, but the number of consecutive night shifts did not affect the levels of self-reported sleepiness among Danish police officers. It is important to keep in mind that working many consecutive night shifts has been recognized as a health hazard in the long run. Thus, when planning night shift work, and more specifically the number of consecutive night shifts, it is important to take into consideration all relevant health hazards as well as performance effects, not just sleepiness.

## Figures and Tables

**Table 1:** Description of 73 participating police officers.

	n	%	Mean (SD)	Range
Age (years)	73		38 (10)	25–62
Tenure within the police force (years)	73		11 (10)	1–32
Night shift work experience (years)			11 (8)	1–30
<3 years	16	22		
3–10 years	27	38		
>10 years	29	40		
Physical activity				
Physically inactive	3	4		
Light physical activity	13	18		
Moderate physical activity	34	47		
High physical activity	22	31		
Self-rated overall health				
Excellent	22	31		
Quite good	34	47		
Good	14	19		
Less good	2	3		
Poor	0	0		
General job satisfaction				
Very dissatisfied	3	4		
Dissatisfied	0	0		
Satisfied	29	40		
Very satisfied	41	56		
Diurnal type				
Morning	10	14		
More morning than evening	10	14		
More evening than morning	38	52		
Evening	15	21		

**Table 2:** Sleep onset and mean total sleep time (TST) for every day in the three different work schedules. TST (incl. naps) was assessed by actigraphy.

Shift Schedule	Night Shift or Recovery Day	Sleep Onset (hh:mm)		TST (h:mm)	
		Mean	(SD)	Mean	(SD)
2+2	Night shift	08:15	(1:34)	05:46	(1:37)
2+2	Night shift	08:16	(1:49)	05:04	(1:30)
2+2	Recovery day	23:35	(1:15)	07:00	(1:50)
2+2	Recovery day	23:41	(1:20)	06:46	(1:28)
4+4	Night shift	08:10	(1:46)	05:58	(1:44)
4+4	Night shift	08:01	(1:17)	05:54	(1:32)
4+4	Night shift	07:56	(0:54)	05:57	(1:21)
4+4	Night shift	08:12	(1:48)	05:08	(1:18)
4+4	Recovery day	00:04	(2:13)	06:53	(1:37)
4+4	Recovery day	23:56	(1:40)	06:31	(1:33)
4+4	Recovery day	23:17	(1:08)	06:50	(1:29)
4+4	Recovery day	23:13	(1:03)	06:48	(1:43)
7+7	Night shift	07:38	(1:24)	06:01	(1:27)
7+7	Night shift	07:52	(0:44)	05:57	(1:22)
7+7	Night shift	07:56	(1:12)	05:47	(1:19)
7+7	Night shift	07:55	(1:17)	06:00	(1:24)
7+7	Night shift	07:49	(1:47)	06:04	(1:21)
7+7	Night shift	07:53	(1:14)	05:50	(1:28)
7+7	Night shift	08:19	(1:27)	04:44	(1:39)
7+7	Recovery day	23:48	(1:44)	06:27	(1:59)
7+7	Recovery day	23:44	(1:57)	07:03	(1:58)
7+7	Recovery day	23:33	(1:21)	06:45	(1:47)
7+7	Recovery day	23:53	(2:01)	06:20	(1:33)
7+7	Recovery day	23:52	(1:41)	06:47	(1:44)
7+7	Recovery day	23:46	(1:37)	06:48	(1:32)
7+7	Recovery day	23:29	(1:13)	07:09	(1:57)

**Table 3:** Mean KSS and number of observations for the different work schedules.

Work Schedule	2+2 Day		4+4 Day		7+7 Day	
Time of Day	Mean (SD)	n	Mean (SD)	n	Mean (SD)	n
07	4.1(2.15)	33	3.0 (1.9)	46	4.0 (2.3)	38
11	2.7 (1.7)	61	2.4 (1.9)	60	2.7 (2.2)	65
15	3.0 (2.1)	64	2.8 (2.8)	65	2.7 (2.2)	68
19	3.1 (2.0)	65	2.8 (1.9)	65	2.8 (2.0)	67
23	4.5 (2.4)	44	4.9 (2.1)	36	5.1 (2.3)	50
03	6.2 (3.0)	5	6.0 (4.2)	2	7.0 (2.0)	5
**Work Schedule**	**2+2 Night**		**4+4 Night**		**7+7 Night**	
**Time of Day**	**Mean (SD)**	**n**	**Mean (SD)**	**n**	**Mean (SD)**	**n**
07	6.0 (2.4)	38	5.7 (2.4)	40	5.5 (2.6)	44
11	5.0 (3.4)	7	3.5 (0.7)	2	6.2 (3.0)	10
15	3.7 (2.0)	53	4.0 (2.5)	45	4.5 (2.3)	51
19	3.3 (2.0)	63	2.6 (1.8)	67	3.5 (2.4)	68
23	3.2 (1.9)	64	2.5 (1.8)	64	3.2 (2.2)	65
03	4.3 (2.4)	62	3.6 (2.3)	59	3.9 (2.4)	62

## Data Availability

The data presented in this study are available on request from the corresponding author. The data are not publicly available due to privacy considerations.
